# 14-3-3 Proteins Are Involved in BR-Induced Ray Petal Elongation in *Gerbera hybrida*

**DOI:** 10.3389/fpls.2021.718091

**Published:** 2021-08-04

**Authors:** Xiaohui Lin, Shina Huang, Gan Huang, Yanbo Chen, Xiaojing Wang, Yaqin Wang

**Affiliations:** ^1^Guangdong Provincial Key Laboratory of Biotechnology for Plant Development, School of Life Sciences, South China Normal University, Guangzhou, China; ^2^College of Landscape Architecture and Art, Henan Agricultural University, Zhengzhou, China

**Keywords:** 14-3-3 protein, ray petal elongation, cell elongation, BR, *Gerbera hybrida*

## Abstract

14-3-3 proteins play a major role in the regulation of primary metabolism, protein transport, ion channel activity, signal transduction and biotic/abiotic stress responses. However, their involvement in petal growth and development is largely unknown. Here, we identified and characterized the expression patterns of seven genes of the 14-3-3 family in gerbera. While none of the genes showed any tissue or developmental specificity of spatiotemporal expression, all seven predicted proteins have the nine α-helices typical of 14-3-3 proteins. Following treatment with brassinolide, an endogenous brassinosteroid, the Gh14-3-3 genes displayed various response patterns; for example, *Gh14-3-3b* and *Gh14-3*-*3f* reached their highest expression level at early (2 h) and late (24 h) timepoints, respectively. Further study revealed that overexpression of *Gh14-3-3b* or *Gh14-3-3f* promoted cell elongation, leading to an increase in ray petal length. By contrast, silencing of *Gh14-3-3b* or *Gh14-3-3f* inhibited petal elongation, which was eliminated partly by brassinolide. Correspondingly, the expression of petal elongation-related and brassinosteroid signaling-related genes was modified in transgenic petals. Taken together, our research suggests that Gh14-3-3b and Gh14-3-3f are positive regulators of brassinosteroid-induced ray petal elongation and thus provides novel insights into the molecular mechanism of petal growth and development.

## Introduction

14-3-3 proteins, with a molecular weight of 25∼32 kDa, are a class of highly conserved, acidic, soluble proteins that are present in almost all eukaryotes (Ferl, 1996; Mhawech, 2005). Each 14-3-3 isoform contains nine α-helices and shows a high degree of similarity to other 14-3-3 members (Aitken, 2006). As bridge proteins, they participate widely in the regulation of various physiological processes (including metabolism, hormone signaling and stress response) by interacting with numerous clients, such as metabolic enzymes, signaling proteins and transcription factors (Silva et al., 2001; Chevalier et al., 2009; Zhou et al., 2015; Li M. et al., 2016; Keicher et al., 2017; Camoni et al., 2018). Generally, 14-3-3 proteins, which are encoded by multiple genes in most species (Denison et al., 2011), bind to their clients via two sequence motifs (RSXpSXP and RXXXpSXP) to activate or inhibit the activity of their target proteins (Sehnke et al., 2002). The first plant 14-3-3 protein identified was cloned from maize (de Vetten et al., 1992). In Arabidopsis, there are thirteen functionally expressed *14-3-3* genes (Chevalier et al., 2009), while tobacco has seventeen potential isoforms, rather more than the eight genes in rice and seven in cotton (Konagaya et al., 2004; Chen et al., 2006; Zhou et al., 2015). According to their gene structure, 14-3-3 proteins can be divided into two groups, termed ε and non-ε (DeLille et al., 2001; Konagaya et al., 2004; Chen et al., 2006; Chevalier et al., 2009).

Brassinosteroids (BRs) are plant steroid hormones that play key roles in regulating a variety of physiological processes, including leaf expansion, flowering, senescence, stress resistance and cell expansion and elongation (Clouse et al., 1996; Clouse and Sasse, 1998; Yang et al., 2011; Kim et al., 2012; Kaneko-Suzuki et al., 2018; Oh et al., 2020). BR signal cascades are well characterized in Arabidopsis, in which BZR1 is the key transcription factor, affecting plant growth and development by modulating thousands of BR target genes and interacting with other hormone signaling components (He et al., 2005; Wang et al., 2012; Qiao et al., 2017; Zheng et al., 2019; Zhang et al., 2020). 14-3-3 proteins are also important regulatory components of the BR signaling pathway: they regulate plant growth by anchoring BZR1 in the cytoplasm (Gampala et al., 2007; Ryu et al., 2007; Kim and Wang, 2010). Mutants of the 14-3-3-binding site of BZR1, with a phenotype similar to that of the *bzr1-1D* mutant, show a constitutive BR response and an increase in BZR1 nuclear retention (Gampala et al., 2007; Ryu et al., 2007).

The 14-3-3 proteins involved in BR signaling participate in plant growth and flowering processes by modulating cell differentiation and elongation (Pertl et al., 2010; Zhang et al., 2010; Taoka et al., 2011; Zhou et al., 2015; Kaneko-Suzuki et al., 2018; Minami et al., 2019). Analysis of multiple *14-3-3* mutants revealed their specificity and functional redundancy in primary root elongation under different environmental conditions, in which these genes are positive regulators under control conditions and negative regulators during abiotic stress (van Kleeff et al., 2014). In lily (*Lilium longiflorum*), 14-3-3 proteins were shown to play a role in the germination and elongation of pollen (Pertl et al., 2010). Minami et al. (2019) reported that, during BR-induced hypocotyl elongation, a 14-3-3 protein interacts with the phosphorylated C-terminus, and thereby enhances the catalytic activity, of plasma membrane H^+^-ATPase. In addition, 14-3-3 proteins have a regulatory role in cotton fiber elongation (Zhou et al., 2015). Thus, overexpression of *14-3-3L* promotes fiber elongation in cotton, while gene silencing of *14-3-3L* results in a shortening of cotton fiber length (Zhou et al., 2015). Recently, Zuo et al. (2021) identified eighteen *14-3-3* genes in the apple genome and characterized their expression patterns, suggesting that some of them may participate in the regulation of the flowering process. These results all highlight the importance of 14-3-3 proteins in plant growth.

*Gerbera hybrida*, belonging to the Asteraceae family, is one of the mainstream cut flowers and its commercial and ornamental value depend on petal morphology and color (Bhatia et al., 2009; Mosqueda Frómeta et al., 2017). Thus, it is important to understand the regulatory mechanisms governing gerbera petal morphology. The research team of Prof. Elomaa has focused on the molecular mechanisms of flower development in Asteraceae, including *G. hybrida*, for many years (Kotilainen et al., 1999; Broholm et al., 2008; Tahtiharju et al., 2012; Juntheikki-Palovaara et al., 2014; Zhao et al., 2020; Zhang et al., 2021). Broholm et al. (2008) found that overexpression of *GhCYC2* in gerbera results in conversion of disc florets into ray-like florets with elongated petals, as well as disruption of stamen development. Functional analysis of GhCYC proteins revealed redundant functions of GhCYC2, GhCYC3 and GhCYC4 in regulating ray floret identity and in promoting petal development (Juntheikki-Palovaara et al., 2014). Various hormones (gibberellin, abscisic acid, ethylene, and BRs) are involved in the regulation of late-stage petal development in gerbera (Zhang et al., 2012; Li et al., 2015; Han et al., 2017; Huang et al., 2017, 2020; Ren et al., 2018). Li et al. (2015) found that GA_3_ stimulates petal elongation in gerbera, while ABA inhibits it. Further research showed that GhWIP2, a WIP-type ZFP transcription factor, represses cell expansion during petal and leaf development by modulating crosstalk between gibberellin, abscisic acid and auxin (Ren et al., 2018). Another study found that exogenous brassinolide (BL) treatment can boost the elongation of ray floret petals, whereas BRZ (a BR synthesis inhibitor) reduces petal length (Huang et al., 2017). However, whether 14-3-3 proteins, as one of the BR signaling components, play a regulatory role in BR-induced petal elongation in gerbera, or indeed in any other flowering species, remains unknown.

Here, seven gerbera 14-3-3 genes were identified and their predicted proteins classified. The expression patterns of all seven genes were comprehensively investigated in various tissues and at different developmental stages. Overexpressing two of these genes, *Gh14-3-3b* and *Gh14-3-3f*, in ray florets increased petal length by promoting cell elongation, whereas gene silencing of *Gh14-3-3b* or *Gh14-3-3f* reduced petal growth. Further analysis found that several BR-related genes, such as BZR1 homologs (*GhBEH1* and *GhBEH2*) and petal elongation-associated genes (like *GhEXP1*, *GhEXP3*, *GhEXP10*, *GhXTH1*, and *GhXET*), were modified in transgenic petals. These results demonstrate a positive regulatory role of Gh14-3-3b and Gh14-3-3f in BR-induced ray petal elongation.

## Materials and Methods

### Plant Materials and Growth Conditions

A variety of *G. hybrida* called “Shenzhen No. 5” was used in this work. The plants were cultured under greenhouse conditions at 26/18°C (day/night temperature) with a 16 h light/8 h dark photocycle and a relative humidity of 65∼80%. Three types of floret (ray floret, trans floret, and disc floret) at stage 6, as well as young leaf (leaf from plants transplanted into the soil for 10∼15 days), old leaf (basal leaf of plants transplanted into the soil for 3 months), young root (root of plants transplanted into the soil for 10∼15 days), old root (root of plants transplanted into the soil for 3 months), calyx, scape, and ray florets at different developmental stages, were sampled for quantitative real-time PCR (qRT-PCR) analysis. The development stages of ray florets (S1∼S6, “S” represents “stage”) are defined according to Meng and Wang (2004). Ray florets at stage 3 were used for transient transformation and hormone treatment assays.

### Cloning and Sequence Analysis of *Gh14-3-3* Genes

Using the sequences of *14-3-3* genes in *Arabidopsis thaliana*, BLAST was performed against the transcriptome shotgun assembly database (Accession: PRJNA179026) of *G. hybrida* cultivar “Shenzhen No. 5” (taxid: 18101) (Kuang et al., 2013), and seven *Gh14-3-3* genes were identified. Seven full-length *Gh14-3-3* cDNA sequences were amplified from a gerbera cDNA library by PCR using PrimeSTAR Max Premix (Takara, Cat. No. R045) with specific primers. Alignment of the deduced amino acid sequences with Gh14-3-3 homolog from different species was performed using DNAMAN 6.0. Conserved domain analysis was executed in the Conserved Domain Database^1^. Protein structure prediction was performed with SWISS-MODEL^2^. Phylogenetic analysis was performed in MEGA 6.0 using a neighbor-joining algorithm with 1,000 bootstrap replicates. The primers for the constructs in each experiment and 14-3-3 protein information for various species are listed in Supplementary Table 1.

### RNA Extraction and qRT-PCR Analysis

Total RNA was extracted from the samples using the Easystep^®^ Super Total RNA Extraction Kit (Promega, Code No. LS1040) following the manufacturer’s protocol. First-strand cDNA was synthesized from 1 μg total RNA using the ReverTra Ace qPCR RT Master Mix with gDNA Remover (Toyobo, Code No. FSQ-301). qRT-PCR was performed using RealStar Green Fast Mixture (GenStar, Code No. A301-01). 1 μL cDNA was added as a qPCR template in a total reaction volume of 20 μL. The samples were amplified using the CFX96 Touch^TM^ Real-Time PCR Detection System (Bio-Rad Laboratories, Inc., United States) as follows: melting at 95°C for 2 min and amplification with 40 cycles of 95°C for 5 s and 60°C for 30 s. All analyses used a housekeeping gene (*GhACTIN*, AJ763915) as a normalization control (Kuang et al., 2013). The expression level was calculated according to the 2^–ΔΔCt^ method. The primers used for qRT-PCR are listed in Supplementary Table 1.

### Transient Transformation of Ray Florets

Transient transformation of ray florets was performed as described by Han et al. (2017). Overexpression vectors (C17 and C17-Gh14-3-3b/f) and virus-induced gene silencing (VIGS) vectors (pTRV1, pTRV2, and pTRV2-Gh14-3-3b/f) were transformed into *Agrobacterium tumefaciens* strain C58C1. *A. tumefaciens* were cultured in Luria-Bertani medium containing 75 mg mL^–1^ kanamycin and 50 mg mL^–1^ rifampicin for 24 h at 28°C and then inoculated into 50 mL Luria-Bertani medium containing 20 μM acetosyringone (AS) and 10 mM 2-(N-morpholino) ethanesulfonic acid (MES) and shaken at 28°C overnight. When the absorbance (OD_600_) of *A. tumefaciens* reached approximately 1.5, the cells were resuspended in infiltration buffer (200 μM AS, 10 mM MES, 10 mM MgCl_2_, pH 5.6) to a final optical density at 600 nm (OD_600_) of 1.5. *Agrobacterium tumefaciens* cultures carrying C17-Gh14-3-3b and C17-Gh14-3-3f, and the empty C17 vector as a mock treatment control, were stored at 28°C for 4 h in the dark at room temperature. *Agrobacterium tumefaciens* cultures carrying pTRV2-Gh14-3-3b/f and pTRV1 at a ratio of 1:1 (v/v), and a mixture containing pTRV2/pTRV1 as a mock treatment control, were also stored under the same conditions for 4 h.

Detached ray petals from fresh inflorescences at stage 3 were cleaned, and then immersed in the various resuspension buffers mentioned above under a vacuum of −0.09 MPa for 5 min. After 2 min, the vacuum was slowly released and the petals were rinsed with sterile distilled water (dH_2_O) and placed in sterile Petri dishes with two layers of Whatman filter paper. After incubation at 4°C for 3 days, the transformed petals were grown at 23∼25°C for 9 days at 50∼60% humidity under long-day conditions (16 h light/8 h dark). At least 15 well-grown inflorescences were used for each treatment, and at least three biological replicates were used for each experiment.

### Hormone Treatment of Ray Florets

Our previous study showed that detached petals can develop normally. The result of *in vitro* hormone and inhibitor experiments performed with detached petals were consistent with the result of *in vivo* experiments using intact inflorescences (Li et al., 2015; Huang et al., 2017). In this study, detached ray petals from inflorescences at stage 3 were used for BL treatments as described previously (Huang et al., 2017). Transiently transformed petals were placed in sterile Petri dishes with two layers of Whatman filter paper soaked in 10 μM BL or dH_2_O as control. Subsequently, the petals were cultured at 24∼26°C for 2 days. At least 15 well-grown inflorescences were used for each treatment, and at least three biological replicates were used for each experiment.

### Measurement of Ray Petal and Cell Length

A total of 45 petals were selected to measure their length as previously described (Li et al., 2015). Petals were imaged with a Nikon camera D7200 (Japan) and measured using ImageJ software. To measure the petal cell length and number, the top, middle and basal region of each petal were stained with propidium iodide (0.1 mg mL^–1^) for 5 min. Next, images of adaxial epidermal cells were captured using a confocal laser scanning microscope (LSM710, Carl Zeiss, Germany) and more than 50 cells were analyzed using ImageJ software. At least three biological replicates were used for each observation. The elongation rate was calculated according Han et al. (2017).

### Statistical Analysis

The data were analyzed with SPSS (version 13.0; IBM Corp., Armonk, NY, United States). Statistical significance between samples was investigated by Duncan’s new multiple range test. The data are presented as mean ± standard error (SE). Different lowercase letters above the bars or line charts indicate significantly different groups: ^∗^*P* < 0.05, ^∗∗^*P* < 0.01.

## Results

### Isolation and Characterization of *Gh14-3-3* Genes in Gerbera

**FIGURE 1:**
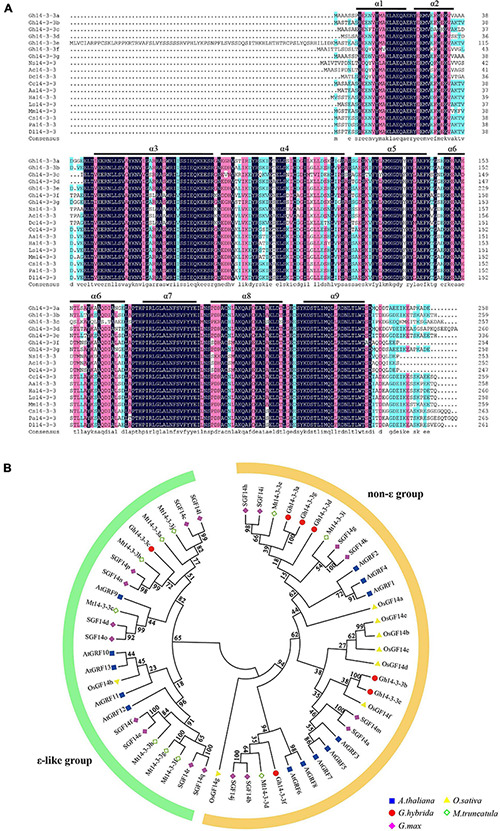
Sequence analysis and phylogenetic tree of Gh14-3-3 proteins. **(A)** Amino acid alignment of Gh14-3-3 proteins with those from other plants. Ns14-3-3 (KAA8528342.1) *Nyssa sinensis*; Ac14-3-3 (PSR99833.1) *Actinidia chinensis*; Dc14-3-3 (XP_017252199.1) *Daucus carota*; Cc14-3-3 (XP_024978074.1) *Cynara cardunculus*; Aa14-3-3 (PWA87588.1) *Artemisia annua*; Ha14-3-3 (XP_022009462.1) *Helianthus annuus*; Ls14-3-3 (XP_023753461.1) *Lactuca sativa*; Mm14-3-3 (KAD2805724.1) *Mikania micrantha*; Cs14-3-3 (XP_028084704.1) *Camellia sinensis*; Pa14-3-3 (PON78199.1) *Parasponia andersonii*; Dl14-3-3 (ACK76233.1) *Dimocarpus longan*. The regions of the nine conserved antiparallel α-helices (α1∼α9) are underlined in black. **(B)** Phylogenetic relationship showing two groups of 14-3-3 proteins in the five species. The minimum evolution tree was constructed using MEGA 6.0 from 1,000 bootstrap replicates. Protein designations consist of the prefixes *Arabidopsis thaliana* (At, blue squares), *Oryza sativa* (Os, yellow triangles), *Gerbera hybrida* (Gh, Red circles), *Medicago truncatula* (Mt, green rhombus), and *Glycine max* (Gm, pink rhombus). Detailed information for 14-3-3s from these plant species are listed in Supplementary Table 1.

To identify *14-3-3* genes in gerbera, we performed a tBLASTn search against the gerbera transcriptome data using 14-3-3 protein sequences from Arabidopsis as queries (Kuang et al., 2013). Seven putative *Gh14-3-3* genes (*Gh14-3-3a*, *Gh14-3-3b*, *Gh14-3-3c*, *Gh14-3-3d*, *Gh14-3-3e*, *Gh14-3-3f*, and *Gh14-3-3g*) were identified. As shown in Figure 1, sequence analysis predicted that the seven *Gh14-3-3* genes encode 258, 259, 257, 261, 336, 254, and 258 amino acids, respectively. Sequence alignment of the gerbera protein sequences showed a high degree of identity, >65%, with the 14-3-3 proteins of *Helianthus annuus*, *Lactuca sativa*, and *Cynara scolymus*. All seven gerbera sequences contained the nine antiparallel α-helices that are highly conserved among 14-3-3 proteins (Figure 1A). These results, together with the conserved domain analysis shown in Supplementary Figure 1, revealed that the seven gerbera genes belong to the 14-3-3 family. Phylogenetic tree analysis showed that these seven sequences could be divided into either the ε group or the non-ε group of 14-3-3 proteins (Figure 1B). Gh14-3-3c is located in the ε branch of the tree, while the other six Gh14-3-3 proteins belong to the non-ε group.

### Dimerization Patterns of Gh14-3-3 Proteins

**FIGURE 2:**
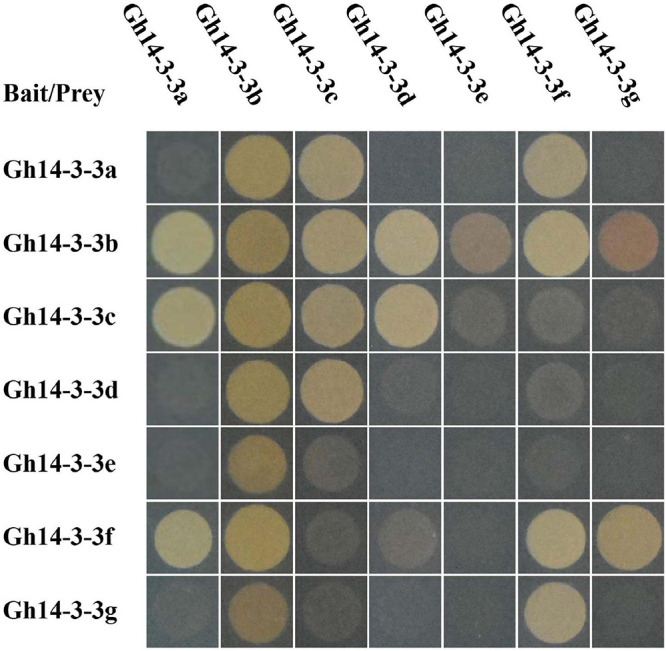
Specific interactions among Gh14-3-3 proteins. pGADT7-largeT7/pGBKT7-53 and pGADT7-largeT7/pGBKT7-laminC were used as positive and negative controls, respectively. The original figure is shown in Supplementary Figure 2.

The 14-3-3 proteins usually exist as homo- or heterodimers (Mhawech, 2005). To investigate protein-protein interactions among the seven Gh14-3-3s, a yeast two-hybrid assay was performed. The results showed that only three proteins (Gh14-3-3b, Gh14-3-3c, and Gh14-3-3f) can form homodimers, while the other protein interaction patterns varied. For example, Gh14-3-3b formed heterodimers with the remaining six Gh14-3-3 proteins, while Gh14-3-3e only interacted with Gh14-3-3b. The other five Gh14-3-3 proteins showed a variety of different dimerization behaviors (Figure 2 and Supplementary Figure 2). These results suggest that Gh14-3-3 protein interaction patterns vary according to isoform.

### Spatiotemporal Expression Patterns and Response of *Gh14-3-3* Genes to BR

**FIGURE 3:**
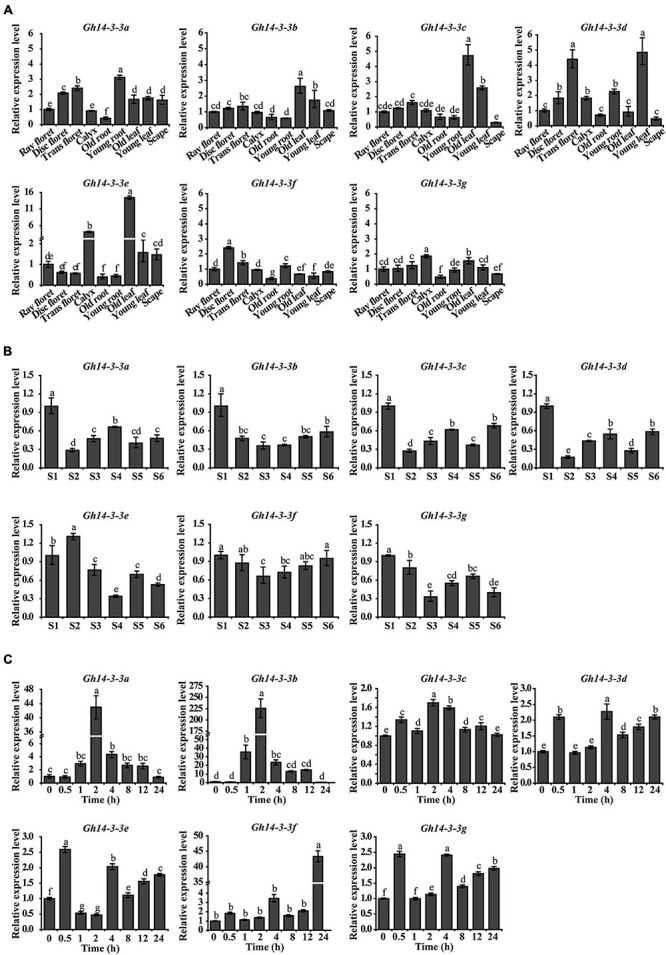
The spatiotemporal expression pattern of *Gh14-3-3* genes. **(A)** The expression pattern of *Gh14-3-3* genes in gerbera tissues and organs. Relative mRNA level of the *Gh14-3-3* genes in gerbera tissues (ray floret, disc floret, trans floret, calyx, old root, young root, old leaf, young leaf, and scape) were detected by qRT-PCR. *GhACTIN* (AJ763915) is the reference gene (Kuang et al., 2013). Gene expression levels were set to 1 in ray floret. **(B)** The expression of *Gh14-3-3* genes during different growth stages (S1∼S6, “S” represents “stage”) of ray floret in *G. bybrida*. The development stages of ray florets were defined according to Meng and Wang (2004). Gene expression levels were set to 1 in S1 ray floret petals. **(C)** The expression level of *Gh14-3-3* genes in ray floret of *G. bybrida* under BL treatments. The expression levels of *Gh14-3-3s* in the ray floret of gerbera were detected within 0∼24 h after BL treatment. Gene expression levels were set to 1 in “0 h” and were calculated using the 2^–ΔΔCt^ method. Values were the means ± SE from three biological replicates.

To explore the spatiotemporal expression patterns of the seven *Gh14-3-3* family members in gerbera, qRT-PCR was performed. We first analyzed their expression in different tissues and found that each gene was expressed in various organs or tissues (Figure 3A). The highest expression levels appeared in young root, young leaf, disc floret, calyx and old leaf, while the lowest expression levels were mostly observed in old root. The different expression profiles in different tissues imply functional diversity in the *Gh14-3-3* gene family.

We next evaluated the expression pattern of *Gh14-3-3* genes during ray floret developmental stages (S1∼S6, “S” represents “stage”) in gerbera. As ray floret petals developed, the expression levels of these genes changed in different ways (Figure 3B). However, some patterns were comparable: for example, *Gh14-3-3b* and *Gh14-3-3f* showed a similar expression pattern, such that their transcription levels declined from the highest level in S1 to the lowest level in S3, followed by a gradual increase. Similarly, the transcript abundance of three genes (*Gh14-3-3a*, *Gh14-3-3c*, and *Gh14-3-3d*) dropped to the lowest level from S1 to S2, and then fluctuated in a related manner. In addition, *Gh14-3-3e* and *Gh14-3-3g* showed the highest expression levels in S2 and S1, and the lowest expression levels in S4 and S3, respectively. These results suggest that the expression of *Gh14-3-3* genes is developmentally regulated in petal cells of gerbera.

14-3-3 proteins play an essential role in the BR signaling pathway (Gampala et al., 2007; Ryu et al., 2007). To determine whether the expression of any of the seven *Gh14-3-3* genes responds to BR, the transcript levels of these genes were evaluated following BL treatment. As shown in Figure 3C, *Gh14-3-3a* and *Gh14-3-3b* shared the same expression profile: both genes began to respond at 1 h after BL treatment, rising to the highest expression level at 2 h, and then gradually decreasing to the lowest level at 24 h. Specifically, *Gh14-3-3b* had the highest peak value (225) among all seven members in response to BL. Three other genes (*Gh14-3-3d*, *Gh14-3-3e*, and *Gh14-3-3g*) had a similar response pattern to BR with two comparable response peaks (2.0∼2.5) at 0.5 h and 4 h. The expression level of *Gh14-3-3f* increased over the study period to a maximum at 24 h, while *Gh14-3-3c* expression varied slightly within a narrow range in response to BR. These results indicate that all members of the *Gh14-3-3* gene family responded to BR, with *Gh14-3-3a* and *Gh14-3-3b* both reaching the highest expression level at an early stage (2 h) after treatment and *Gh14-3-3f* at a late stage (24 h).

### *Gh14-3-3b* and *Gh14-3-3f* Promote Ray Petal Elongation in Gerbera

Based on the above results, it is clear that *Gh14-3-3b* and *Gh14-3-3f* have the lowest expression level in S3 (the onset of cell elongation), compared to other developmental stages. The two genes reached their highest expression levels at early (2 h) and late stages (24 h) in response to BR, respectively. Thus, we chose to analyze the roles of *Gh14-3-3b* and *Gh14-3-3f* in ray petal elongation by transient overexpression and VIGS assays.

**FIGURE 4:**
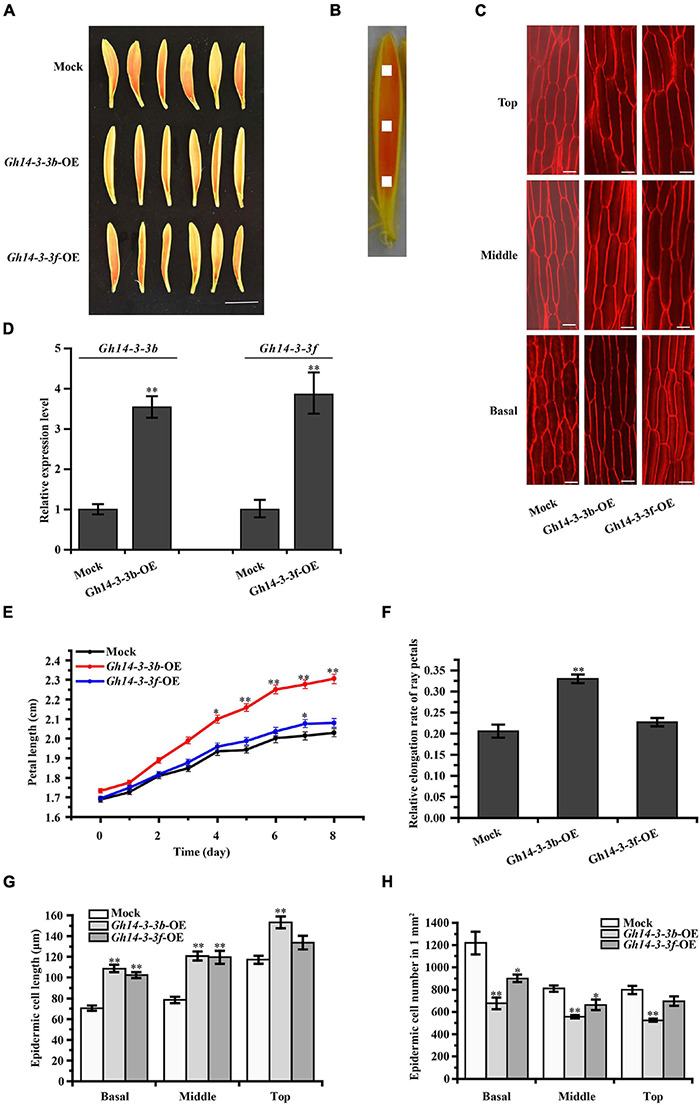
Petal elongation of ray florets were promoted by the overexpression of *Gh14-3-3b* and *Gh14-3-3f*. **(A)** Petal phenotype of the *Gh14-3-3b*, *Gh14-3-3f* transient overexpression and Mock (*Gh14-3-3b*-OE, *Gh14-3-3f*-OE, Mock) after 8 days. Bar = 1 cm. **(B)** Schematic diagram of the petal. The picture shows basal, middle, top regions of ray floret petals. The white area (1 mm^2^) shows the measured region for cell length. **(C)** The confocal microscope image of epidermal cells in top, middle and basal regions of petals. Bar = 20 μm. **(D)** The expression analysis of *Gh14-3-3b* and *Gh14-3-3f* in the Mock and transgenic ray petals by qRT-PCR. The length **(E)** and relative elongation rates **(F)** of mock, *Gh14-3-3b*-OE and *Gh14-3-3f*-OE petals (*n* = 45). The cell length **(G)** and cell number **(H)** in top, middle, and basal regions of transgenic ray petals. At least three biological replicates were used for each observation.

Overexpression of *Gh14-3-3b* significantly promoted petal length and elongation rate, while *Gh14-3-3f*-OE petals showed slightly increased petal length and elongation rate (Figures 4A,D–F). The elongation rate was 0.33 ± 0.01 in *Gh14-3-3b*-OE and 0.23 ± 0.01 in *Gh14-3-3f*-OE petals, which corresponds to increases of 57% and 10%, respectively, compared with an elongation rate of 0.21 ± 0.02 in the mock experiment (Figure 4F). To determine whether overexpression of *Gh14-3-3b* and *Gh14-3-3f* regulates petal length by promoting petal epidermal cell length, both cell length and number in the top, middle and basal regions of ray petals were measured (Figures 4B,C). The epidermal cell lengths of *Gh14-3-3b*-OE petals were markedly longer than in the mock controls in all three regions (Figures 4C,G). In addition, the epidermal cell numbers in *Gh14-3-3b*-OE petals were much smaller than in mock-treated equivalents (Figure 4H). For *Gh14-3-3f*-OE ray florets, epidermal cell lengths in the basal and middle regions were significantly longer than in the mock, while epidermal cell numbers in the basal and middle regions were lower. The results suggest that *Gh14-3-3b* and *Gh14-3-3f* promote ray petal elongation by regulating cell elongation.

**FIGURE 5:**
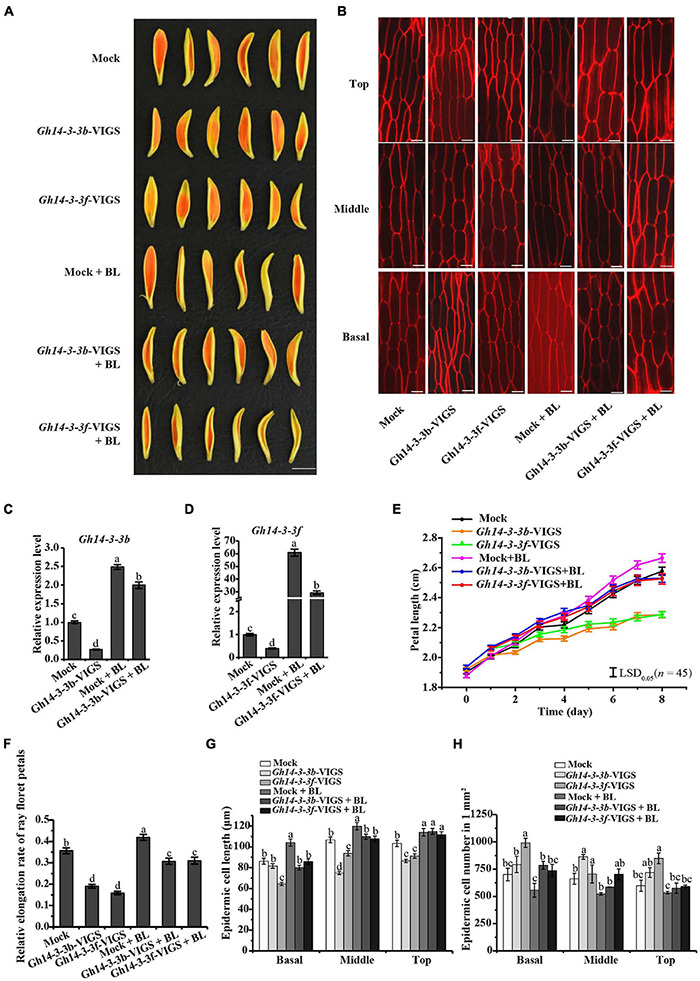
Petal elongation of ray florets was inhibited by transient silencing of *Gh14-3-3b* and *Gh14-3-3f*. **(A)** Petal phenotype of Mock, *Gh14-3-3b* transient silencing, *Gh14-3-3f* transient silencing, and those treated with 10 μM BL (Mock, *Gh14-3-3b*-VIGS, *Gh14-3-3f*-VIGS, Mock + BL, *Gh14-3-3b*-VIGS + BL, *Gh14-3-3f*-VIGS + BL). Bar = 1 cm. **(B)** The confocal microscope image of epidermal cells in top, middle and basal regions of petals. Bar = 20 μm. **(C)** The relative expression level of *Gh14-3-3b* in Mock, *Gh14-3-3b*-OE, Mock + BL and *Gh14-3-3b*-VIGS + BL. **(D)** The relative expression level of *Gh14-3-3f* in Mock, *Gh14-3-3f*-OE, Mock + BL, and *Gh14-3-3f*-VIGS + BL. The length **(E)** and relative elongation rate **(F)** of Mock, *Gh14-3-3b*-VIGS, *Gh14-3-3f*-VIGS, Mock + BL, *Gh14-3-3b*-VIGS + BL, and *Gh14-3-3f*-VIGS + BL petals (*n* = 45). The elongation rate was calculated at day 8. The cell length **(G)** and cell number **(H)** in top, middle, and basal regions in transgenic ray petals. At least three biological replicates were used for each observation.

We further confirmed the role of *Gh14-3-3b* and *Gh14-3-3f* in ray petal elongation using the VIGS system. As shown in Figures 5A,E,F, gene silencing of both *Gh14-3-3b* and *Gh14-3-3f* significantly shortened the length and elongation rate of ray petals, relative to the mock. However, exogenous BL treatment eliminated partly this repression (Figures 5A,E). Gene silencing of *Gh14-3-3b* and *Gh14-3-3f* also reduced the epidermal cell lengths and boosted cell numbers compared to the mock, while BR reversed this phenotype to some extent (Figures 5B,G,H). In addition, we analyzed the expression of *Gh14-3-3b* and *Gh14-3-3f* in *Gh14-3-3b*-VIGS and *Gh14-3-3f*-VIGS petals without and with BL treatment (Figures 5C,D). Surprisingly, BL induced the expression of both *Gh14-3-3b* and *Gh14-3-3f* to levels that were approximately 10-fold and 60-fold greater, respectively, than those of *Gh14-3-3b*-VIGS and *Gh14-3-3f*-VIGS petals. Taken together, these results suggest that *Gh14-3-3b* and *Gh14-3-3f* promote BR-induced ray petal elongation.

### Expression of Genes Involved in BR Signaling and Petal Elongation Is Altered in *Gh14-3-3b* and *Gh14-3-3f* Transgenic Ray Petals

**FIGURE 6:**
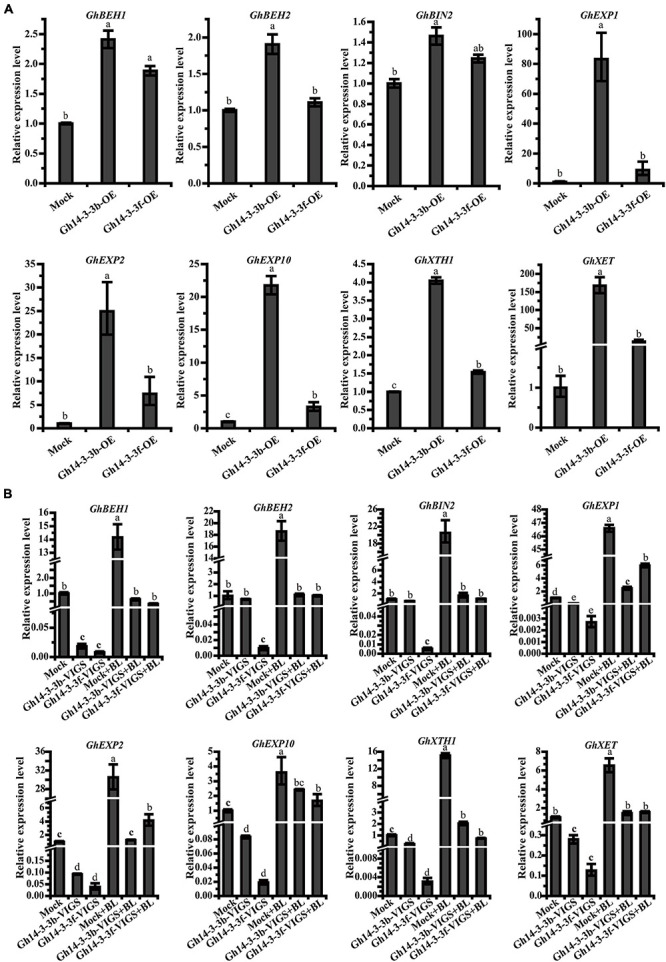
The expression level of related genes in *Gh14-3-3b/f*-OE ray floret petals **(A)** and *Gh14-3-3b/f*-VIGS ray floret petals **(B)**. Gene expression levels were calculated using the 2^–ΔΔCt^ method. The accession numbers of related genes in GenBank are: *GhBEH1* (GACN01037487.1), *GhBEH2* (GACN01006390.1), *GhBIN2* (GACN01003499.1), *GhEXP1* (GACN01041978.1), *GhEXP2* (GACN01002748.1), *GhEXP10* (GACN01039741.1), *GhXTH1* (GACN01007419.1), and *GhXET* (GACN01007419.1).

To investigate the mechanism by which *Gh14-3-3b* and *Gh14-3-3f* regulate ray petal elongation, the expression levels of genes involved in BR signaling and petal elongation were determined. As shown in Figure 6A, the expression of BR signaling genes (*GhBEH1*, *GhBEH2*, and *GhBIN2*) was significantly increased in *Gh14-3-3b*-OE petals, but only marginally upregulated in *Gh14-3-3f*-OE petals. The expression levels of genes involved in petal elongation (*GhEXP1*, *GhEXP2*, *GhEXP10*, *GhXTH1*, and *GhXET*) were markedly boosted in *Gh14-3-3b*-OE and *Gh14-3-3f*-OE petals, especially in the former (Figure 6A).

On the other hand, the expression levels of the above genes showed, in many cases, a declining trend in *Gh14-3-3b*-VIGS and *Gh14-3-3f*-VIGS petals. In the *Gh14-3-3f*-VIGS petals, the expression of all eight genes was significantly reduced, compared to the mock (Figure 6B), while in *Gh14-3-3b*-VIGS petals, the expression of six genes (*GhBEH1*, *GhEXP1*, *GhEXP2*, *GhEXP10*, *GhXTH1*, and *GhXET*) was markedly downregulated, with two genes, *GhBEH2* and *GhBIN2*, showing only a slightly decline. Notably, the expression of all eight genes was enhanced, albeit to different degrees, following BL treatment. These results suggest that *Gh14-3-3b* and *Gh14-3-3f* regulate BR-induced ray petal elongation by modulating genes associated with BR signaling and petal development.

## Discussion

### Gh14-3-3 Proteins, Which Fall Into Two Groups, May Possess Functional Diversity

Since the first plant 14-3-3 protein was cloned from maize (de Vetten et al., 1992), researchers have identified eight 14-3-3 proteins in rice (Chen et al., 2006; Denison et al., 2011), 18 in apple (Zuo et al., 2021), nine in common bean (Li M. et al., 2016) and seven in cotton (Zhou et al., 2015). In the present study, seven 14-3-3 isoforms were identified in the gerbera transcriptome. Sequence analysis showed that all isoforms share the conserved nine α-helical regions typical of the 14-3-3 family and have 254∼336 amino acids (Figure 1A). Phylogenetics classified the seven gerbera 14-3-3 proteins into two groups, the ε and non-ε groups, consistent with similar groupings in Arabidopsis, rice, and banana (Yaffe et al., 1997; Chevalier et al., 2009; Li M. et al., 2016). Furthermore, they show a high degree of identity with 14-3-3 proteins in *Helianthus annuus* and *Lactuca sativa*, both of which belong to Asteraceae family, hinting that these proteins have similar functions across the Asteraceae (Figure 1A).

Previous studies revealed that 14-3-3 proteins can form homodimers or, instead, can form heterodimers with different isoforms, which promotes functional diversity (Liu et al., 1995; Xiao et al., 1995; Aghazadeh et al., 2015; Ormancey et al., 2017). Each isoform displays a different propensity to dimerize with others, depending on the highly variable amino acid sequences in their N-terminal helices (Liu et al., 1995; Xiao et al., 1995). Consistent with this, the sequences of the N-terminal helices of gerbera 14-3-3 proteins are less well conserved than their C-terminal helices (Figure 1A) and only three of the gerbera proteins (Gh14-3-3b, Gh14-3-3c, and Gh14-3-3f) form homodimers (Figure 2 and Supplementary Figure 2). Among the seven Gh14-3-3 proteins, the number of heterodimers formed ranged from one for Gh14-3-3e to six for Gh14-3-3b, which demonstrates the selectivity of each isoform in protein-protein interactions.

Wilson et al. (2016) summarized the regulatory mechanisms of 14-3-3 proteins in several plants during the development of multiple organs, including seedling, leaf, root, flower, and developing seed. We found that the seven 14-3-3 isoforms are expressed in various organs in gerbera to different extents (Figure 3A). This implies a functional diversity among all seven members, similar to the 14-3-3 proteins of other species (Chen et al., 2006; Yao et al., 2007). In addition, we surveyed the expression patterns of the *Gh14-3-3* genes during ray floret developmental phases (S1∼S6) (Figure 3B). As development progresses through stages S1 to S6, the *Gh14-3-3* genes are expressed in various patterns. For example, *Gh14-3-3b* and *Gh14-3-3f* display a trend of earlier decrease and later increase from S1∼S6, and they both have the lowest expression level at S3 (Figure 3B). These genes also differ in their response to exogenous BL treatment (Figure 3C): *Gh14-3-3b* responds rapidly and reaches its highest expression level in the early phase of the experiment (2 h), while *Gh14-3-3f* shows a slow response pattern. These results suggest functional diversity of the *Gh14-3-3* genes during BR-induced gerbera growth and development (Chen et al., 2006; Zhou et al., 2015; Zuo et al., 2021).

### Gh14-3-3b and Gh14-3-3f Play a Positive Regulatory Role in BR-Induced Ray Petal Elongation

The roles of 14-3-3 proteins in plant growth and development have been reported (Zhou et al., 2015; Huang et al., 2018). At14-3-3λ and At14-3-3K are involved in shade-induced hypocotyl elongation via PHYTOCHROME-INTERACTING FACTOR 7 (Huang et al., 2018). In cotton, 14-3-3 proteins are involved in cotton fiber elongation by regulating GhBZR1 protein, which binds to the promoters of genes involved in fiber development (Zhou et al., 2015). In the present study, overexpression of *Gh14-3-3b* and *Gh14-3-3f* in gerbera increased the length of ray petals, whereas gene silencing of *Gh14-3-3b* and *Gh14-3-3f* shortened petal length (Figures 4E, 5E). Confocal images showed that this may be achieved by regulating epidermal cell length in petals (Figures 4G, 5G). Moreover, the qRT-PCR results revealed that the expression of genes involved in BR signaling (*GhBEH1*, *GhBEH2*, and *GhBIN2*) is enhanced in *Gh14-3-3b*-OE and *Gh14-3-3f*-OE petals and inhibited in *Gh14-3-3b*-VIGS and *Gh14-3-3f*-VIGS petals (Figures 6A,B). BL treatment promotes petal length in *Gh14-3-3b*-VIGS and *Gh14-3-3f*-VIGS petals, and enhances the expression of *GhBEH1* in *Gh14-3-3b*-VIGS petals as well as the expressions of *GhBEH1*, *GhBEH2*, and *GhBIN2* in *Gh14-3-3f*-VIGS petals (Figures 5E, 6B). These results suggest that Gh14-3-3b and Gh14-3-3f play a role in BR-induced ray petal elongation. In a previous study, BL was shown to promote ray petal elongation and *GhBEH1* expression in gerbera (Huang et al., 2017). In Arabidopsis, AtBZR1 is one of the most important transcription factors in BR signaling and promotes cell elongation in response to BR (Oh et al., 2012; Chaiwanon and Wang, 2015). Therefore, it is possible that Gh14-3-3b and Gh14-3-3f modulate ray petal elongation by regulating GhBEH1 and GhBEH2. The specific molecular mechanisms will be investigated further in future studies.

Expansins (EXPs), xyloglucan endotransglycosylases (XETs), and xylan transferases/hydrolases (XTHs) are involved in cell wall remodeling and cell elongation (Takeda et al., 2002; Harada et al., 2011; Che et al., 2016; Li Y. et al., 2016; Rao and Dixon, 2017; Ackerman-Lavert and Savaldi-Goldstein, 2020). Two *XTH* genes (*DcXTH2* and *DcXTH3*) and two expansin genes (*DcEXPA1* and *DcEXPA2*) are associated with petal growth and development during flower opening in carnation (Harada et al., 2011). *OsEXPA10* is expressed in the root tips and is necessary for cell elongation in rice (Che et al., 2016). Additionally, the expression of some *XTH* and *EXP* genes is markedly enhanced by BL treatment in Arabidopsis (Park et al., 2010) and soybean (Rao and Dixon, 2017). Our previous study also showed that BR promotes ray petal elongation and initiates the expression of a number of genes, including those encoding two putative cell wall proteins (Huang et al., 2017). In this study, the expression of a number of petal elongation-related genes (including *GhEXP1*, *GhEXP2*, *GhEXP10*, *GhXET*, and *GhXEH*) were altered in *Gh14-3-3b*-OE and *Gh14-3-3f*-OE, as well as *Gh14-3-3b*-VIGS and *Gh14-3-3f*-VIGS petals (Figure 6B). This suggests that Gh14-3-3b and Gh14-3-3f modulate petal elongation-related gene transcription, thereby mediating petal cell elongation and petal development.

As one of the mainstream cut flowers, gerbera has a high demand in the market. However, it has fewer flower types compared to chrysanthemum. Thus, obtaining a variety of flower types is one of the main goals of gerbera breeding, which requires an understanding of the regulatory mechanism of gerbera flower development. In this study, seven Gh14-3-3 protein genes were identified and their expression patterns were characterized. These genes share a conserved structure, but display different dimerization patterns, which implies they are functionally diverse. Transient transformation assays demonstrated that Gh14-3-3b and Gh14-3-3f play a positive regulatory role in BR-induced ray petal elongation. Thus, as well as providing novel insights into the role of 14-3-3 proteins in ray petal elongation, this study also highlights a number of candidate genes for flower type breeding of gerbera.

## Data Availability Statement

The original contributions presented in the study are included in the article/Supplementary Material, further inquiries can be directed to the corresponding author.

## Author Contributions

XL carried out the experiments, drafted the manuscript, and revised manuscript. SH conducted the experiments, analyzed the data, and prepared the figures. GH participated in part of the experiments. YC and XW revised the manuscript. YW conceived the study, participated in its design, and revised the manuscript. All authors read and approved the final manuscript.

## Conflict of Interest

The authors declare that the research was conducted in the absence of any commercial or financial relationships that could be construed as a potential conflict of interest.

## Publisher’s Note

All claims expressed in this article are solely those of the authors and do not necessarily represent those of their affiliated organizations, or those of the publisher, the editors and the reviewers. Any product that may be evaluated in this article, or claim that may be made by its manufacturer, is not guaranteed or endorsed by the publisher.

## Footnotes

1
https://www.ncbi.nlm.nih.gov/Structure/cdd/wrpsb.cgi

2
https://swissmodel.expasy.org/interactive

## Supplementary Material

Supplementary Figure 1 Conserved domains analysis of seven Gh14-3-3s in the Conserved Domain Database.

Supplementary Figure 2 The interactions among Gh14-3-3 proteins. *Gh14-3-3s* were introduced into pGADT7-AD and pGBKT7-BD. pGADT7-largeT7/pGBKT7-53 and pGADT7-largeT7/pGBKT7-laminC were used as positive and negative controls, respectively.

Supplementary Table 1 Primers used in this study and amino acid sequences of 14-3-3s in different species.
