# Supplementary figures and images for: 14-3-3 Proteins Are Involved in BR-Induced Ray Petal Elongation in Gerbera hybrida
Source: Front Plant Sci. 2021 Aug 4;12:718091. doi: 10.3389/fpls.2021.718091 (PMC8371339; doi:10.3389/fpls.2021.718091)

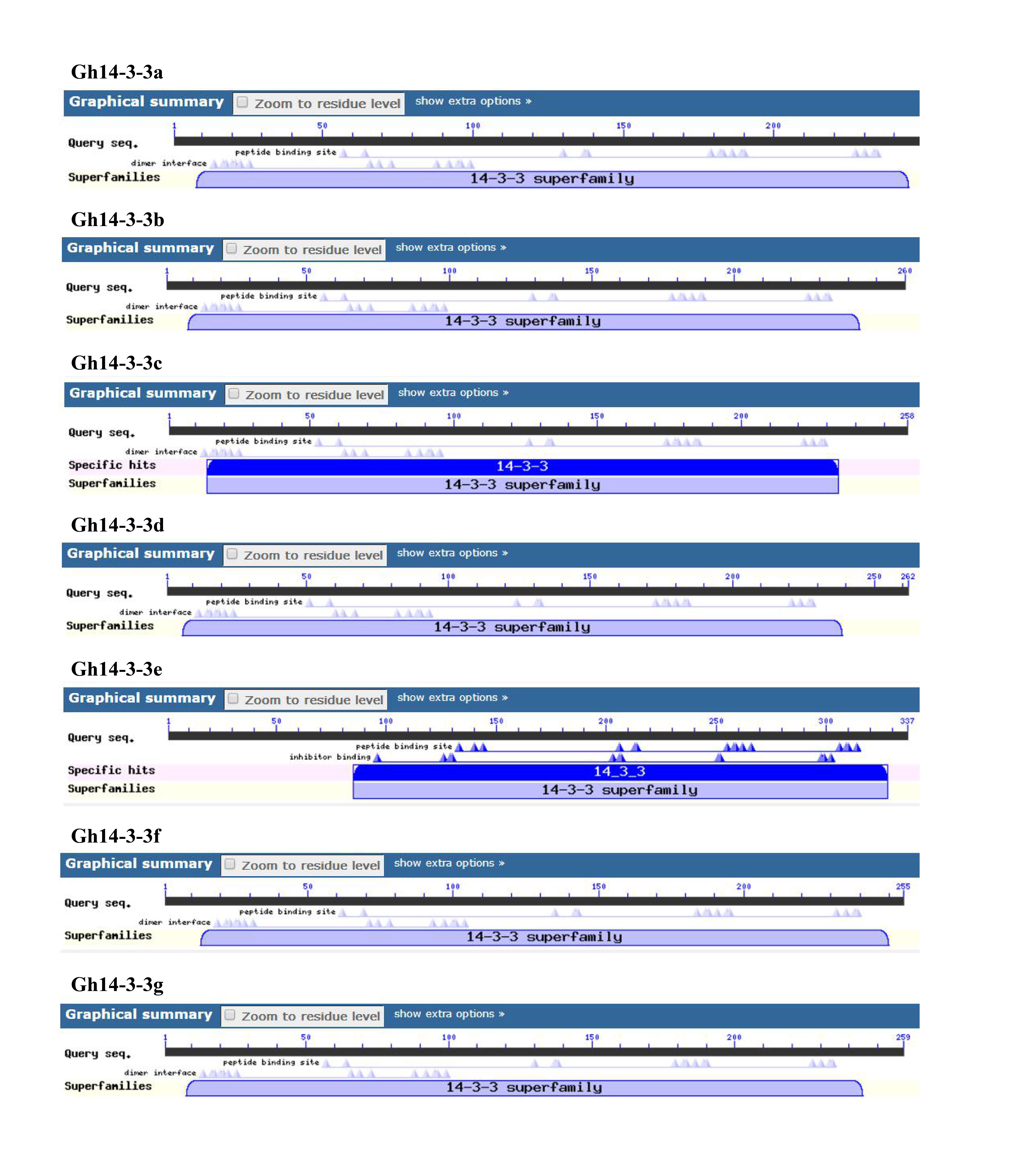

Supplement: Supplementary Figure 1 — Conserved domains analysis of seven Gh14-3-3s in the Conserved Domain Database. [file Image_1.JPEG]

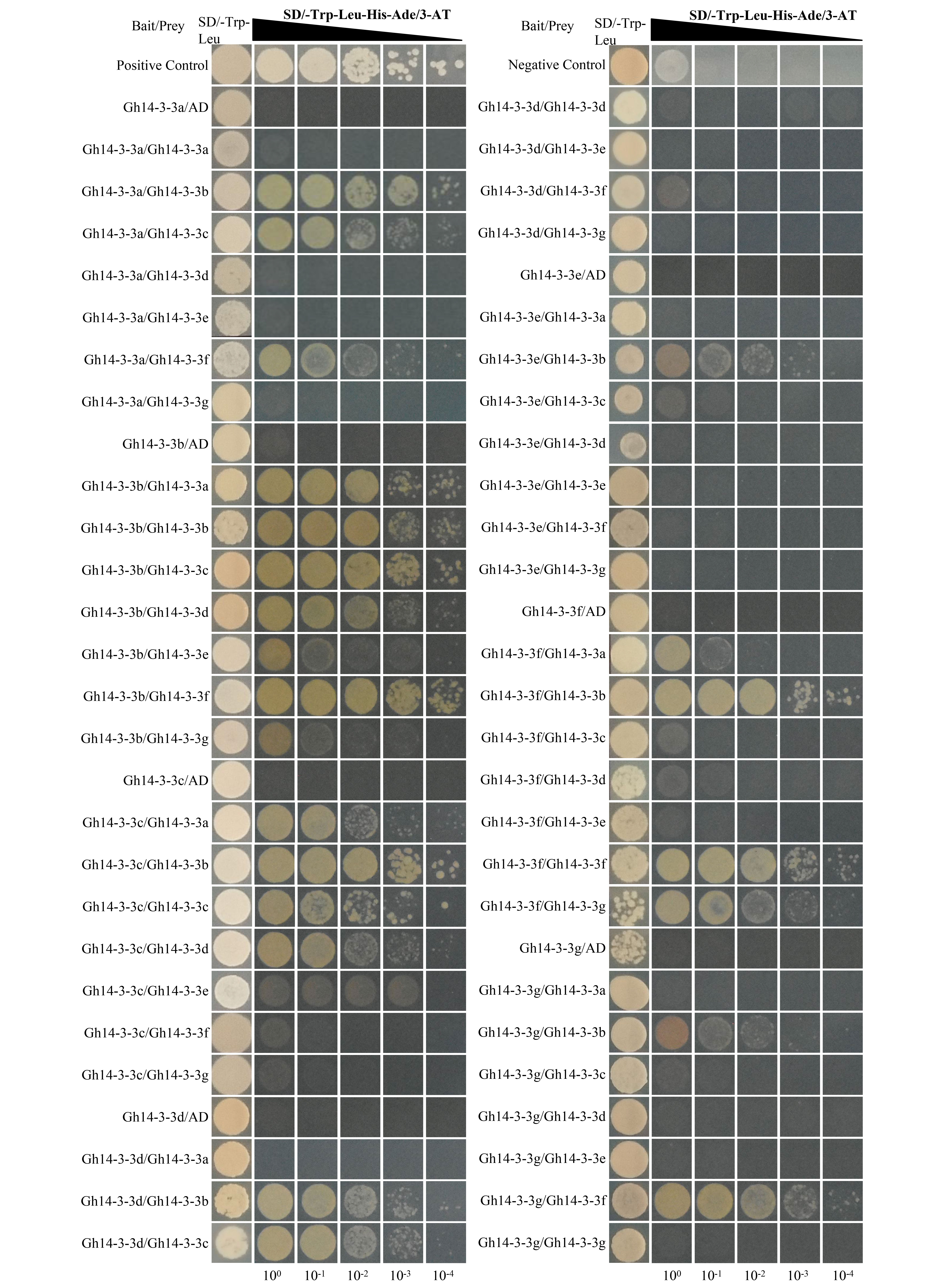

Supplement: Supplementary Figure 2 — The interactions among Gh14-3-3 proteins. Gh14-3-3s were introduced into pGADT7-AD and pGBKT7-BD. pGADT7-largeT7/pGBKT7-53 and pGADT7-largeT7/pGBKT7-laminC were used as positive and negative controls, respectively. [file Image_2.JPEG]
